# Positive predictive value of the prostate imaging reporting and data system combined with single related indicators in prostate cancer across different prostate zones

**DOI:** 10.3389/fonc.2026.1661267

**Published:** 2026-02-04

**Authors:** XiaFeng Shen, LuYao Yang, ShiWei Wang, JianLiang Shen

**Affiliations:** 1 Department of Radiology, The First Affiliated Hospital of Zhejiang Chinese Medical University (Zhejiang Provincial Hospital of Chinese Medicine), Hangzhou, China; 2 Zhejiang Chinese Medical University, Hangzhou, China

**Keywords:** positive predictive value, prostate cancer, prostate imaging reporting and data system, PSA density, zoning

## Abstract

**Introduction:**

This study aimed to evaluate the positive predictive value (PPV) of the Prostate Imaging Reporting and Data System (PI-RADS) combined with single related indicators in diagnosing prostate cancer (PCa) across different prostate zones.

**Methods:**

Patients with complete clinical data who underwent prostate magnetic resonance imaging from January 2019 to October 2024 were retrospectively analyzed. PI-RADS was used for diagnosis, zoning, and grading, with 533 cases scoring ≥3. PPVs for PCa across different prostate zones were calculated by combining age, prostate-specific antigen (PSA), PSA density (PSAd), and prostate volume. Differences between non-PCa and PCa groups were compared using independent sample *t*- and rank-sum tests. Diagnostic efficacy was assessed using area under the curve (AUC) values for receiver operating characteristic curves. Univariate logistic regression analysis was used to identify factors associated with malignant pathology.

**Results:**

The PPV for PI-RADS scores 3–5 was 20.6% (33/160), 61.1% (159/260), and 80.5% (91/113), respectively. PPVs for PCa across peripheral, transitional, and multi-zones were 78.6% (96/122), 35.2% (114/323), and 82.9% (73/88), respectively. Age, PSA, PSAd, and prostate volume significantly differed between the non-PCa and PCa groups, with AUC values of 0.629, 0.709, 0.809, and 0.703, respectively, and were significantly associated with malignant pathology (*P*< 0.001, univariate logistic regression analysis).

**Conclusion:**

Combining the PI-RADS with other clinical indicators effectively enhanced its initially low PPV for transitional zone lesions, particularly when the PSAd was ≥0.15 ng/mL^2^ or the PSA was >10 ng/mL.

## 1 Introduction

Multiparametric magnetic resonance imaging (mpMRI) is recognized as the optimal imaging modality for diagnosing prostate cancer (PCa), aiding in diagnosis, staging, and active surveillance (1–4). The Prostate Imaging Reporting and Data System (PI-RADS) serves as a standardized framework for PCa management, communication, and quality assurance in multicenter studies (5–7). Despite its widespread clinical use, the PI-RADS exhibits certain limitations, such as the subjectivity of image interpretation and inter-observer differences. Radiologists with differing levels of experience may attribute different PI-RADS scores for the same lesion (inter-observer reliability), and even the same radiologist may report different readings at different times (intra-observer reliability) (8, 9).

Screening using related indicators, such as prostate-specific antigen (PSA), facilitates early PCa detection; however, their specificity remains low (10, 11). PI-RADS and clinical indicators such as PSA provide information derived from different modalities; therefore, a combination of these two modalities may improve accuracy in diagnosing PCa.

A higher positive predictive value (PPV) can be used to rule in clinically significant PCa, which helps to avoid unnecessary biopsies. Therefore, this study aimed to retrospectively analyze the PPV performance of the PI-RADS combined with single related indicators in diagnosing PCa. We considered the location of the PCa to account for shortcomings in previous studies that did not account for the prostate zones.

## 2 Materials and methods

### 2.1 Clinical data

This study was approved by the Ethics Committee of The First Affiliated Hospital of Zhejiang Chinese Medical University (Zhejiang Provincial Hospital of Chinese Medicine; IRB No. 2022-K-081-01), and informed consent was obtained from all patients.

We retrospectively analyzed the data of 2,037 patients who underwent prostate magnetic resonance imaging (MRI) at The First Affiliated Hospital of Zhejiang Chinese Medical University (Zhejiang Provincial Hospital of Chinese Medicine) from January 2019 to October 2024.

**Figure 1:**
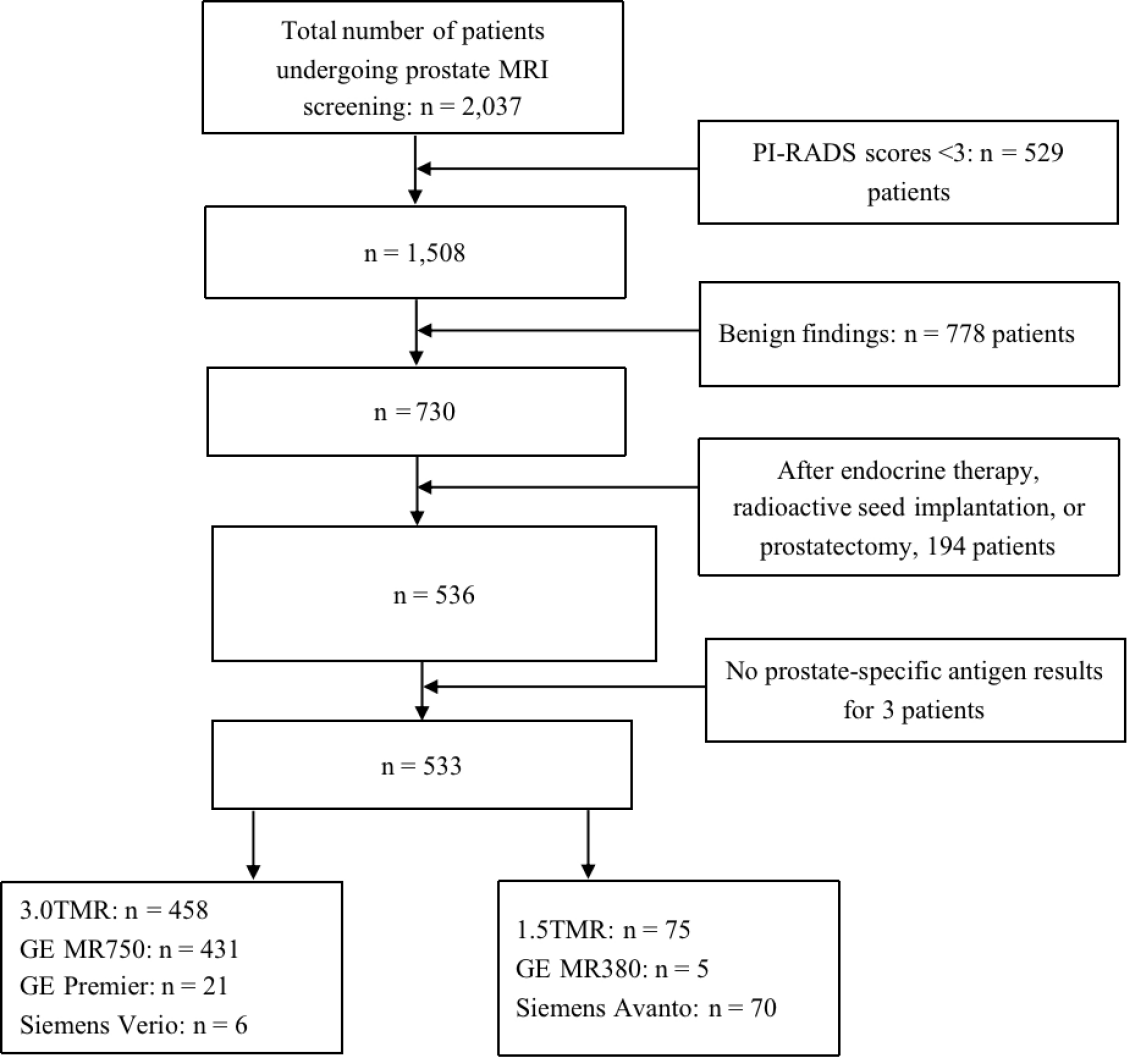
Flowchart of patient inclusion and MRI scanner distribution. MRI, magnetic resonance imaging.

The inclusion criteria comprised patients with complete prostate MRI imaging and clinical data and a PI-RADS score ≥3, and those who underwent prostate biopsy or surgery performed within one month of MRI with definitive pathological results. The magnetic resonance sequence comprised axial T1-weighted imaging (T1WI), axial T2-weighted imaging (T2WI), sagittal or coronal T2WI, and axial diffusion weighted imaging (DWI) sequence. The clinical data included age, PSA, PSA density (PSAd), prostate volume, and pathological results. The exclusion criteria comprised patients with poor MRI quality or significant artifacts; those with MRI scans in which MRI lesion locations could not be matched with the pathological results; and patients who had undergone endocrine therapy, prostate radioactive seed implantation, or prostatectomy prior to MRI. Ultimately, 533 patients were included, with the selected lesion being the one with the highest PI-RADS score per patient (**Figure 1**).

### 2.2 MRI scanning methods

Prostate MRI scans were obtained using 1.5T or 3.0T MRI scanners, including GE Discovery MR750 3.0T, Premier 3.0T, MR380 1.5T (General Electric Company, Boston, MA, USA), Siemens Verio 3.0T, and Siemens Avanto 1.5T (Siemens Healthineers, Erlangen, Germany). Scans utilized abdominal phased-array coils, with patients positioned supine, feet first, covering the prostate and seminal vesicles. Sequences included axial T1WI, axial T2WI, sagittal or coronal T2WI, and axial DWI. Magnetic resonance sequence parameters are shown in **Table 1**.

**Table 1:** Sequence parameters for multiparametric magnetic resonance imaging protocol.

Parameter	T1WI (axial)	T2WI (axial)	T2WI (coronal)	T2WI (sagittal)	DWI (axial)	DCE
TR/TE (ms)	600/12	4000/96	4600/110	4500/110	4000/70	3/1
Slice thickness (mm)	3	3	3	3	3	3
Flip angle	90	90	90	90	90	13
FOV (mm)	160	160	200	200	180	160/200
Matrix	256 × 256	256 × 256	256 × 256	256 × 256	140×140	256 × 256

DCE, dynamic contrast-enhanced; DWI, diffusion-weighted imaging; FOV, field of view; T1WI, T1 weighted imaging; T2WI, T2 weighted imaging.

In total, 412 of 533 patients underwent dynamic contrast-enhanced (DCE) imaging with gadopentetate dimeglumine (Beijing Beilu Pharmaceutical Co., Ltd., Beijing, China) injected via the cubital vein (dose, 0.2 mL/kg; rate, 2.0 mL/s). The total scan time was approximately 25 min (30 min with enhancement).

### 2.3 Image analysis

Two experienced radiologists performed secondary reviews, scoring and zoning lesions using the PI-RADS v2.1 software, after which a consensus was reached. The intraclass correlation coefficient (ICC) was used to evaluate consistency between the PI-RADS scores assigned by the two radiologists. The results showed that consistency between the two physicians was good (ICC = 0.827). In cases of disagreement, a senior radiologist (an associate professor or higher) from the abdominal imaging group provided the final evaluation. When multiple regions are involved, lesions were categorized into peripheral zone (PZ), transitional zone (TZ), and multi-zone (MZ) groups based on T2WI. PZ lesions were scored mainly based on DWI and apparent diffusion coefficient (ADC) maps, with a high signal on DWI and a low signal on ADC maps indicating positive results. TZ lesions were mainly scored on T2WI, with poorly defined low-to-medium signal nodules often indicating positive results. Left-right, anteroposterior, and superoinferior diameters was measured by two radiologists on T2WI, and Equation 1 was used to calculate the prostate volume.

(1) volume=left−right×anteroposterior×superoinferior×0.52

Equation 1 Prostate volume formula and then averaged. One radiologist matched the pathological lesions to MRI findings, ensuring correspondence with the PI-RADS scores.

### 2.4 Histopathology

A uniform puncture technique was used for all patients. Transrectal ultrasound-guided prostate biopsy was performed by a senior ultrasound physician using a 12+X needle pattern: 12 needles for routine sampling of the inner and outer glands bilaterally, plus X needles targeting MRI- or ultrasound-identified regions of interest. PCa was graded by a professional pathologist using the Gleason scoring system (5 levels, 10 points). Non-cancerous benign findings included benign prostatic hyperplasia, acute/chronic prostatitis, high/low-grade prostatic intraepithelial neoplasia, and atypical small acinar proliferation.

### 2.5 Statistical analyses

Statistical analyses were conducted using SPSS 25.0 software (SPSS Inc., San Diego, CA, USA). Normally distributed continuous data are expressed as mean ± standard deviation and were compared using independent sample *t*-tests. Non-normally distributed data are presented as median (interquartile range) and were compared using rank-sum tests. Receiver operating characteristic (ROC) curves were plotted to calculate the area under the curve (AUC) to assess the diagnostic efficacy of age, PSA, PSAd, and prostate volume for PCa. Univariate logistic regression analysis identified factors associated with malignant pathology. A *P*-value<0.05 indicated statistical significance.

PPV was calculated for the PI-RADS across different prostate zones and grades. Based on clinical guidelines and literature, PPV was also computed for lesions exceeding risk thresholds as follows: (i) PI-RADS + age group (age ≥65 years) (12); (ii) PI-RADS + PSA(A) group (PSA ≥4 ng/mL), PI-RADS + PSA(B) group (PSA 4–10 ng/mL grey zone) (13), and PI-RADS + PSA(C) group (PSA >10 ng/mL); (iii) PI-RADS + PSAd group (PSAd ≥0.15 ng/mL²) (14); and (iv) PI-RADS + volume group (prostate volume<40 mL) (15).

## 3 Results

### 3.1 Clinical data

**Figure 2:**
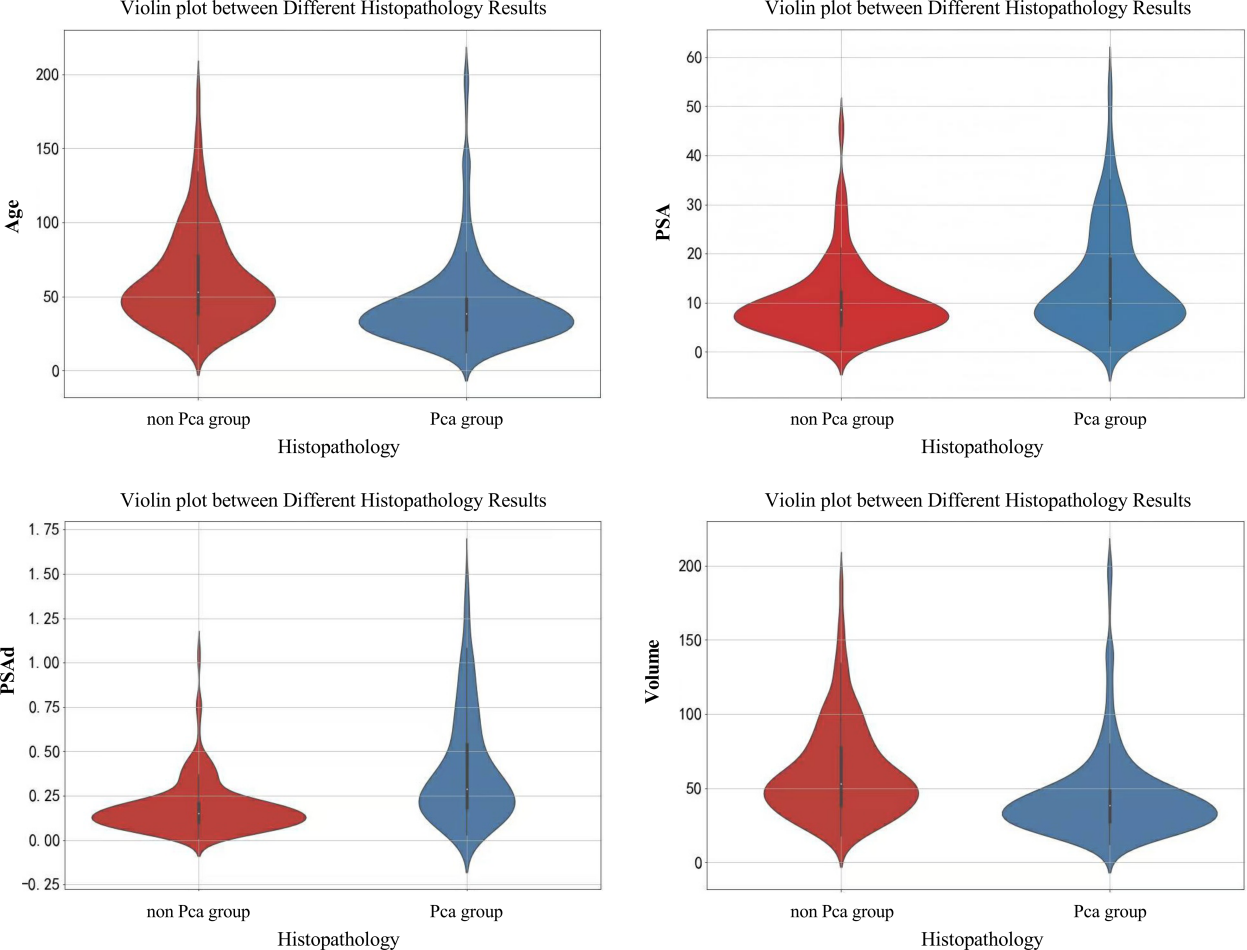
Violin plots between different histopathology results.

The mean age of the included patients was 70.39 ± 8.59 years, with PSA levels of 10.32 (15.83) ng/mL, PSAd of 0.22 (0.41) ng/mL², and prostate volume of 44.10 (32.28) mL. The mean age, PSA level, PSAd, and prostate volume were 68.45 ± 8.20 years, 8.61 (7.08) ng/mL, 0.15 (0.11) ng/mL², and 53.05 (39.21) mL for the non-PCa group and 72.10 ± 8.58 years, 14.97 (33.12) ng/mL, 0.41 (1.02) ng/mL², and 38.38 (21.15) mL for the PCa group, respectively (**Supplementary Table S1**). These differences between the groups were statistically significant (*t* = 5.01, *z* = 8.32, *z* = 8.08, and *z* = 12.31, respectively; all *P*< 0.001; **Figure 2**). Moreover, univariate logistic regression analysis indicated that age, PSA, PSAd, and prostate volume were associated with malignant pathology in the TZ group (**Table 2**).

**Table 2:** Univariate logistic regression analysis of indicators and malignant pathology across prostate zones.

Zoning	OR (95% CI)				*P*-value			
	Age	PSA	PSAd	Volume	Age	PSA	PSAd	Volume
PZ	1.07 (1.01–1.12)	1.03 (0.99–1.07)	4.08 (1.01–16.59)	0.98 (0.96–0.99)	0.013	0.114	0.050	0.046
TZ	1.07 (1.04–1.10)	1.04 (1.02–1.07)	48.07 (12.07–191.44)	0.98 (0.97–0.99)	<0.001	<0.001	<0.001	<0.001
MZ	1.05 (0.99–1.12)	1.10 (1.02–1.19)	508.45 (4.74–54549.66)	0.99 (0.98–1.01)	0.125	0.010	0.009	0.397
Total	1.05 (1.03–1.08)	1.05 (1.04–1.07)	46.76 (16.74–130.64)	0.98 (0.97–0.99)	<0.001	<0.001	<0.001	<0.001

CI, confidence interval; OR, odds ratio; PSA, prostate-specific antigen; PSAd, prostate-specific antigen density.

**Figure 3:**
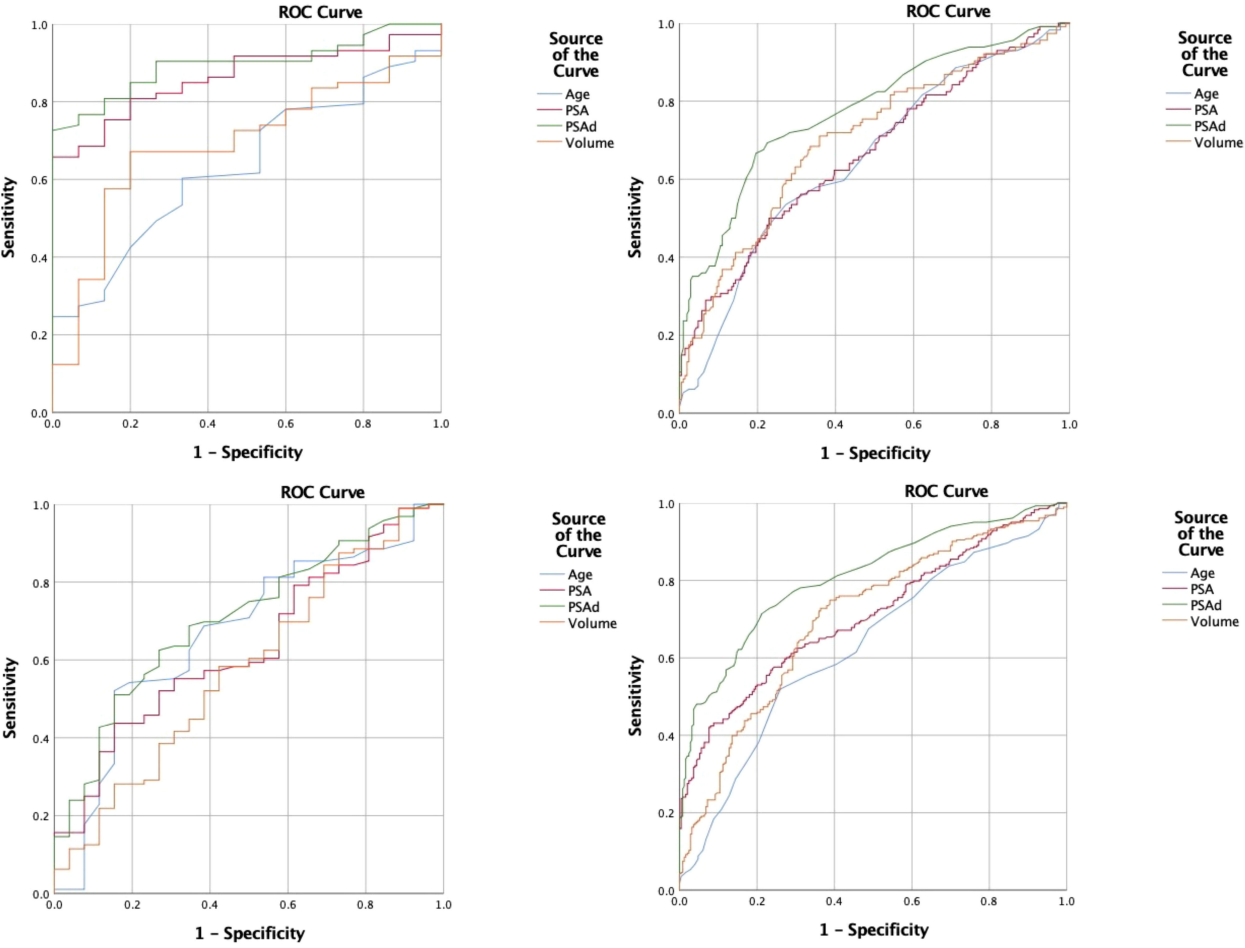
ROC curve of related indicators to predict benign and malignant lesions. ROC, receiver operating characteristic.

Using histological pathology as the gold standard, ROC curves were plotted with age, PSA, PSAd, and prostate volume as variables. The AUC of age was 0.629, sensitivity was 51.90%, and specificity was 74.00%. For PSA, the AUC was 0.709, sensitivity was 42.40%, and specificity was 92.00%. The AUC of PSAd was 0.809, sensitivity was 71.40%, and specificity was 78.80%. The AUC of prostate volume was 0.703, with a sensitivity of 63.60% and a specificity of 72.80% (**Figure 3**; **Supplementary Table S2**).

### 3.2 Pathological results

In total, 283 lesions were pathologically confirmed as PCa, and 250 lesions as non-PCa. Among these, 235 cases were clinically significant PCa (Gleason score ≥ 3 + 4), and 48 were non-clinically significant PCa. There were 96 cases of PCa in the PZ, 114 cases of PCa in the TZ, and 73 cases of PCa accumulated in multiple zones (detailed information and Gleason scores are shown in **Table 3**).

**Table 3:** Gleason score distribution of pathologically confirmed lesions.

Group	Lesion	3 + 3	3 + 4	4 + 3	≥8	Unknown
Total	283	48 (17.0%)	44 (15.6%)	43 (15.2%)	126 (44.5%)	22 (7.8%)
PZ	96	13	10	17	45	11
TZ	114	32	28	14	32	8
MZ	73	3	6	12	49	3

MZ, multi-zone; PZ, peripheral zone; TZ, transitional zone.

### 3.3 PPV results

The overall PPV of the PI-RADS was 53.1% (283/533). For PI-RADS scores 3, 4, and 5, the PPV was 20.6% (33/160), 61.1% (159/260), and 80.5% (91/113), respectively. The PPV for the TZ group (35.2%) was lower than those of the PZ (78.6%) and MZ groups (82.9%). When combined with the related indicators, the PPV for the PI-RADS + age, PI-RADS + PSA(A), PI-RADS + PSAd, and PI-RADS + volume groups was 57.6% (237/411), 54.9% (274/499), 64.6% (247/382), and 70.2% (156/222), respectively (**Supplementary Table S3**).

## 4 Discussion

PI-RADS scores are critical for guiding prostate biopsy decisions (16), typically prompting biopsy when PI-scores are ≥4 and PCa is not yet confirmed, leading to a high likelihood of biopsy. In this study, we identified a PPV of 67.0% for PI-RADS scores ≥4 at our center, indicating that approximately 30% of patients had undergone unnecessary biopsies, incurring additional costs.

Similar to the results at our center, Westphalen et al. (17) analyzed the PI-RADS PPV of 26 different imaging centers. When the PI-RADS score was ≥4, the PPV ranged from approximately 26% to 75%, and the average PPV was approximately 49% (95% confidence interval [CI] 40–58%), with large PPV variability among the different imaging centers. The difference in PPV may be related to variances in protocol parameters of MRI in different imaging centers, as well as the subjective image interpretation by different radiologists. At our center, the percentage of PI-RADS PPV scores of 3 (moderate risk) was 20.6% (33/160). Similar to our results, Westphalen et al. reported a finding of 15% (95% CI 11–19%), suggesting limited net benefit from biopsy. However, the cost of missing a diagnosis is unacceptable, necessitating selective biopsy guided by additional clinical indicators. Furthermore, the PPV study of Westphalen et al. did not consider the location of PCa, whereas our study considered the different regions of the prostate gland to obtain more accurate information.

The second edition of the PI-RADS v2.0 (2015) (18) established distinctive scoring criteria for PZ and TZ lesions, further refined in the PI-RADS v2.1 (2019) (18–20), reflecting differing imaging characteristics. Our study findings indicated that, with the same PI-RADS score, lesions in the TZ (35.2%) had a significantly lower PPV than those in the PZ (78.6%) or MZ (82.9%).

TZ often contains varying numbers of hyperplastic nodules, some atypical and potentially cancerous (21), making definitive characterization challenging amidst multiple nodules. Consequently, radiologists frequently recommend biopsy for defensive purposes, likely contributing to the low PPV in TZ. Therefore, in this study, we investigated combining the PI-RADS with clinical indicators to enhance the PPV for PI-RADS 3 and TZ lesions.

Our differential analysis revealed statistically significant differences between the PCa and non-PCa groups for age, PSA, PSAd, and prostate volume (*t* = 5.01, *z* = 8.32, *z* = 8.08, and *z* = 12.31, respectively; all *P*< 0.001) (22–24). The AUC values indicated good diagnostic efficacy for PSA, PSAd, and prostate volume. Moreover, combining the PI-RADS with these indicators improved PPV. Specifically, combining the PI-RADS with PSA resulted in a decrease in the PPV by 12.5% in the 4–10 ng/mL grey zone (PI-RADS + PSA(B) group), suggesting many non-PCa lesions. However, it increased to 66.4% when the PSA was >10 ng/mL (C group). Combining the PI-RADS with the PSAd yielded a PPV of 64.6%, similar to the C group, with logistic regression analysis indicating the strongest association with PCa (OR 46.76). These results suggest that PSAd is a valuable reference indicator. Moreover, the combination of the PI-RADS with prostate volume achieved a PPV of 70.2%; however, its reference value is limited owing to a low odds ratio (0.98) and a lack of a clinically accepted volume threshold. For example, the use of a volume of 40 mL reduced the sample size from 533 to 222, introducing bias. Combining the PI-RADS with PSA (>10 ng/mL) and PSAd increased the PPV for PI-RADS lesions with score 3 from 20% to approximately 30% and TZ lesions from 35% to approximately 50%, demonstrating a marked improvement.

This study had some limitations. The sample was sourced from a single center and only included patients who underwent biopsy; patients who did not undergo biopsy were excluded, which might have introduced bias. Variability in the MRI field strength (3.0T and 1.5T) and a lack of DCE sequences in some cases may have affected the PI-RADS scoring. Moreover, we did not distinguish between clinically significant and non-clinically significant PCa. Finally, potential false negatives in some pathology results were observed, negatively affecting the PPV.

In conclusion, the PI-RADS exhibited low PPV for TZ and PI-RADS 3 lesions, but in combination with related indicators, it enhanced the PPV across prostate zones and grades, particularly when the PSAd was ≥0.15 ng/mL² or the PSA was >10 ng/mL. However, the PPV for TZ and PI-RADS 3 lesions remained lower than that for the other groups. These study results highlight the importance of PPV and biopsy decision-making for TZ and PI-RADS 3 lesions. Supporting this, the deep-learning model constructed by Cai et al. (25) showed enhanced diagnostic performance and may reduce observer error and provide more reliable results. These findings encourage further research and technological advancements to improve PI-RADS PPV and reduce unnecessary biopsies.

## Data Availability

The original contributions presented in the study are included in the article/**Supplementary Material**. Further inquiries can be directed to the corresponding author.
