# Supplementary material for: Positive predictive value of the prostate imaging reporting and data system combined with single related indicators in prostate cancer across different prostate zones
Source: Front Oncol. 2026 Feb 4;16:1661267. doi: 10.3389/fonc.2026.1661267 (PMC12913086; doi:10.3389/fonc.2026.1661267)
Supplement: Supplementary file 1 [file Table1.docx]

Supplemental Table1 Clinical data and lesion distribution of patients

|  |  | n | Age（y） | PSA（ng/ml） | PSAd（ng/ml^2^） | Volume（ml） |
| --- | --- | --- | --- | --- | --- | --- |
| PZ | Total | 122 | 69.86±8.85 | 10.97（19..98） | 0.33（0.61） | 32.27（17.93） |
|  | Non-PCa | 26 | 65.92±10.25 | 9.36（7.56） | 0.21（0.21） | 36.73（24.10） |
|  | PCa | 96 | 70.93±8.17 | 11.88（23.33） | 0.39（0.78） | 31.74（17.49） |
| TZ | Total | 323 | 70.29±8.27 | 9.54（9.24） | 0.18（0.23） | 49.75（35.72） |
|  | Non-PCa | 209 | 68.79±8.01 | 8.61（7.11） | 0.15（0.12） | 54.25（39.07） |
|  | PCa | 114 | 73.04±8.06 | 12.36（17.05） | 0.31（0.51） | 39.94（27.31） |
| MZ | Total | 88 | 71.48±9.37 | 30.26（87.40） | 0.75（2.42） | 43.73（29.32） |
|  | Non-PCa | 15 | 68.07±6.43 | 8.23（6.43） | 0.18（0.12） | 58.27（31.64） |
|  | PCa | 73 | 72.18±9.75 | 43.54（111.42） | 1.03（2.59） | 41.74（29.56） |

PSA, prostate-specific antigen; PSAd, prostate-specific antigen density

Supplemental Table 2 Diagnostic performance of related Indicators in Prostate Cancer Across Different Zones

|  | AUC（95%CI） | P | Cut-off | Sensitivity | Specificity |
| --- | --- | --- | --- | --- | --- |
| Age |  |  |  |  |  |
| Total | 0.629 (0.582-0.677） | <0.001 | 72.50 | 0.519 | 0.740 |
| PZ | 0.668 (0.549-0.786） | 0.009 | 72.50 | 0.521 | 0.846 |
| TZ | 0.651 (0.589-0.714） | <0.001 | 72.50 | 0.535 | 0.727 |
| MZ | 0.635 (0.501-0.769） | 0.102 | 70.5 | 0.603 | 0.667 |
| **PSA** |  |  |  |  |  |
| Total | 0.709 (0.665-0.752) | <0.001 | 21.00 | 0.424 | 0.920 |
| PZ | 0.635 (0.602-0.728） | 0.035 | 13.91 | 0.438 | 0.846 |
| TZ | 0.665 (0.549-0.786） | <0.001 | 12.74 | 0.500 | 0.770 |
| MZ | 0.861 (0.783-0.939） | <0.001 | 27.32 | 0.658 | 1.000 |
| **PSAd** |  |  |  |  |  |
| Total | 0.809 (0.773-0.845) | <0.001 | 0.24 | 0.714 | 0.788 |
| PZ | 0.707 (0.601-0.813） | 0.001 | 0.32 | 0.625 | 0.731 |
| TZ | 0.774 (0.720-0.829） | <0.001 | 0.24 | 0.667 | 0.804 |
| MZ | 0.896 (0.831-0.962） | <0.001 | 0.47 | 0.726 | 1.000 |
| Volume |  |  |  |  |  |
| Total | 0.703 (0.658-0.747) | <0.001 | 46.96 | 0.636 | 0.728 |
| PZ | 0.580 (0.455-0.705） | 0.213 | 35.83 | 0.583 | 0.577 |
| TZ | 0.700 (0.639-0.761） | <0.001 | 48.52 | 0.711 | 0.641 |
| MZ | 0.689 (0.559-0.820） | 0.021 | 48.05 | 0.671 | 0.800 |

ROC, receiver operating characteristic; CI, confidence interval;

Supplemental Table3 PPVs of PI-RADS Across Different Zones

|  | PI-RADS | PI-RADS+Age | PI-RADS+PSA | | | PI-RADS+PSAd | PI-RADS+Volume |
| --- | --- | --- | --- | --- | --- | --- | --- |
|  |  |  | A | B | C |  |  |
| PI-RADS=3 | 20.63 | 24.78 | 22.14 | 19.28 | 26.32 | 30.23 | 38.46 |
| PI-RADS≥3 | 53.10 | 57.66 | 54.91 | 40.54 | 66.43 | 64.66 | 70.27 |
| PI-RADS≥4 | 67.02 | 70.13 | 67.69 | 53.24 | 76.82 | 74.66 | 80.00 |
| **PZ** | 78.69 | 84.78 | 80.17 | 76.00 | 83.33 | 81.90 | 81.48 |
| PI-RADS=3 | 38.46 | 44.44 | 41.67 | 37.50 | 50.00 | 45.45 | 55.56 |
| PI-RADS=4 | 83.53 | 89.39 | 83.95 | 87.18 | 80.95 | 85.92 | 85.71 |
| PI-RADS=5 | 83.33 | 88.24 | 86.96 | 33.33 | 95.00 | 86.96 | 81.25 |
| **TZ** | 35.29 | 40.56 | 36.91 | 27.92 | 46.53 | 47.00 | 54.21 |
| PI-RADS=3 | 18.52 | 23.16 | 19.83 | 18.84 | 21.28 | 26.87 | 32.50 |
| PI-RADS=4 | 44.14 | 47.83 | 44.29 | 36.62 | 52.17 | 53.61 | 61.70 |
| PI-RADS=5 | 58.14 | 61.54 | 59.52 | 28.57 | 75.00 | 66.67 | 80.00 |
| **MZ** | 82.95 | 82.86 | 83.53 | 50.00 | 92.54 | 87.01 | 94.12 |
| PI-RADS=3 | 25.00 | 22.22 | 25.00 | 0.00 | 50.00 | 37.50 | 66.67 |
| PI-RADS=4 | 80.00 | 80.00 | 82.14 | 62.50 | 90.00 | 82.14 | 94.12 |
| PI-RADS=5 | 100.00 | 100.00 | 100.00 | 100.00 | 100.00 | 100.00 | 100.00 |

PPV,positive predictive value
